# Surface Roughness Analysis and Prediction with an Artificial Neural Network Model for Dry Milling of Co–Cr Biomedical Alloys

**DOI:** 10.3390/ma14216361

**Published:** 2021-10-24

**Authors:** Manuela-Roxana Dijmărescu, Bogdan Felician Abaza, Ionelia Voiculescu, Maria-Cristina Dijmărescu, Ion Ciocan

**Affiliations:** 1 Manufacturing Engineering Department, University Politehnica of Bucharest, 313 Splaiul Independentei, 060042 Bucharest, Romania; cnelu59@yahoo.com; 2 Quality Engineering and Industrial Technologies Department, University Politehnica of Bucharest, 313 Splaiul Independentei, 060042 Bucharest, Romania; ioneliav@yahoo.co.uk (I.V.); maria.dijmarescu@upb.ro (M.-C.D.); 3 Romanian Research and Development Institute for Gas Turbines, 220 D Iuliu Maniu Bd, 061126 Bucharest, Romania

**Keywords:** roughness prediction, biomedical alloys machining, Co–28Cr–6Mo, Co–20Cr–15W–10Ni, ANN model, AlTiCrSiN PVD coated tool

## Abstract

The aim of this paper is to conduct an experimental study in order to obtain a roughness (Ra) prediction model for dry end-milling (with an AlTiCrSiN PVD-coated tool) of the Co–28Cr–6Mo and Co–20Cr–15W–10Ni biomedical alloys, a model that can contribute to more quickly obtaining the desired surface quality and shortening the manufacturing process time. An experimental plan based on the central composite design method was adopted to determine the influence of the axial depth of cut, feed per tooth and cutting speed process parameters (input variables) on the Ra surface roughness (response variable) which was recorded after machining for both alloys. To develop the prediction models, statistical techniques were used first and three prediction equations were obtained for each alloy, the best results being achieved using response surface methodology. However, for obtaining a higher accuracy of prediction, ANN models were developed with the help of an application made in LabView for roughness (Ra) prediction. The primary results of this research consist of the Co–28Cr–6Mo and Co–20Cr–15W–10Ni prediction models and the developed application. The modeling results show that the ANN model can predict the surface roughness with high accuracy for the considered Co–Cr alloys.

## 1. Introduction

The most commonly used materials for manufacturing medical implants are stainless steels, titanium alloys and CoCrMo alloys [1,2,3,4].

Co–Cr based alloys were patented in 1913, being initially used for applications requiring outstanding resilience in high-temperature corrosion (aircraft engines) and the first alloys of this class were called Stellites [4,5,6]. Later (1930), other types of Co–Cr alloys such as CoCrMoMn (Vitallium) and CoCrMoNiFeMn (HS-21 CoCrMoNi or HS-25 CoCrNi) were developed [1,4,7,8].

The chemical composition of these alloys has undergone changes over time, depending on the required use characteristics. Consequently, for applications in the medical field the molybdenum content was maintained to ensure grain refinement, increase the mechanical strength of the solid solution and corrosion resistance, tungsten was eliminated due to the carbide forming tendency and the lack of homogeneity of very hard phases, while nickel was added to improve toughness and corrosion resistance [8].

Currently, from the six classes of Cobalt alloys standardized in ISO 5832, four are specific to the orthopedic domain. These alloys are available both as cast and wrought products. The Co–Cr alloys recommended for use in medical applications can be classified in the following alloying systems: CoCrMo alloy, CoCrWNi alloy, CoNiCrMo alloy and CoNiCrMoWFe alloy [9,10,11].

The use of nickel-free CoCr alloys is recommended for permanent implantable medical devices, as Ni can affect cell viability and proliferation, examples of which include wrought Co–20Cr–15W–10Ni and Co–Ni–Cr–Mo–W–Fe [1,12,13]. Cast alloys are characterized by a rough dendritic structure. In cobalt-based alloys, coarse dendrites have a high Co content, while interdendritic areas contain some inter-metallic compounds. This microstructural configuration determines a weaker behavior in subsequent processing, so it is preferable to apply grain refinement processes, such as hot isostatic pressing [4].

The most common processes used for manufacturing metallic parts are cutting processes: turning, milling, grinding, etc. The relative measure that defines how easily a material can be cut is called machinability [11,14]. Cutting process reliability is one of the parameters that defines the machinability of a material [14] and it can be characterized by effective surface quality, chip breaking and cutting tool durability [14,15].

Unlike the expansive knowledge developed on titanium alloys and stainless steel machinability from extended scientific studies, scientific data on Co–Cr alloys and particularly CoCrMo alloys requires further research and experimental studies in order to acquire a complete picture of their machinability behavior [16,17,18,19]. When evaluating and measuring the surface quality of machined parts, surface roughness is an important quality criterion [20,21,22,23,24].

The surface roughness of machined parts is influenced by numerous factors, such as material characteristics, cutting data (depth of cut, feed rate, cutting speed), tool geometry, tool wear, cutting fluids, etc. [17,21,24,25,26]. In the case of medical implants, surface roughness has a significant effect on functionality and biocompatibility, influencing important characteristics of the implants as osseointegration, wettability and bacterial adhesion [3,27,28,29,30,31,32]. The main parameter used to evaluate the surface roughness of a machined surface is the average roughness (Ra) [21,33]. In medical implants, the Ra value is most often situated below 2 μm [30,34,35].

Analyzing the research conducted on Co–Cr alloys used in biomedical applications, primarily focusing on CoCrMo and CoCrWNi alloys, it can be ascertained that most studies examine alloys’ microstructure, microstructural changes, corrosion resistance and mechanical properties [36,37,38,39,40,41,42,43,44,45]. The primary objects of the current research trend are implant functionality and biocompatibility, which are determined by the above-listed characteristics. Despite the existing research focus, the manufacture of medical implants also plays a determining role in terms of functionality and cost, and the research conducted in this area for Co–Cr alloys should be further expanded, particularly concerning the surface roughness that can be obtained by various machining processes, given that surface roughness is not only a surface quality indicator, but also an important factor for evaluating the machining performance and the technological operation cost [21].

Several researchers have proposed approaches that evaluate, predict and/or optimize surface roughness obtained from different machining processes, such as analytical models, multiple regression functions, fuzzy logic, genetic algorithms and artificial neural networks (ANN) [15,20,21,24,33,46,47,48,49,50,51,52,53,54,55,56].

A research investigation performed by Zain et al. [33] indicated that using ANN for Ra prediction gives better results compared to other approaches. For example, while the statistical analysis techniques usually generate good prediction models, sometimes these techniques may not present, with high accuracy, the nonlinear relationship between the cutting parameters and the measurements, while ANN models result in more accurate predictions of the dependent variable than a conventional model because the prediction error is smaller for ANNs [21,33,57].

ANN is a rapid and reliable method broadly used for solving complex problems by using various algorithm architectures [33,58,59]. Consequently, this modeling approach can be used as a prediction instrument for determining nonlinear relationships between input and output results for different manufacturing processes [59].

Considering the fact that the Ra roughness parameter is an important criterion for evaluating the surface quality of a medical implant, its functionality and biocompatibility, and the fact that the actual research developed on Co–Cr alloys machinability still present with unexplored areas, it can be ascertained that the prediction of the surface roughness resulting from mechanical processing of the Co–Cr alloys as Co–28Cr–6Mo and Co–20Cr–15W–10Ni is a subject of current interest, particularly due to the fact that these alloys are constantly used in medicine as well as in other fields, and it is important to study this subject in order to gain more knowledge of this area.

The aim of this study is to analyze the surface quality, in terms of the resulting Ra roughness from the dry end-milling of two Co–Cr based alloys for medical devices (Co–28Cr–6Mo and Co–20Cr–15W–10Ni) using AlTiCrSiN PVD coated tools. For this purpose, an ANN application developed in LabView was used to predict the roughness values. The input parameters considered for the experiment and the Ra prediction were axial depth of cut a_p_ (mm), feed per tooth f_z_ (mm/tooth) and cutting speed v_c_ (m/min). The importance and novelty of this study results from the need to establish processing parameter values that permit the faster achievement of a desired surface quality for medical devices manufactured from Co–Cr alloys and, implicitly, to allow for the shortening of the manufacturing process time.

## 2. Materials and Methods

The materials that are the subject of this research study are two Co–Cr based alloys used in medical applications: a CoCrMo alloy (Co–28Cr–6Mo) with a high content of Chromium used in the medical practice for manufacturing implants for dentistry and orthopedic applications, and a CoCrWNi (Co–20Cr–15W–10Ni) alloy used in the medical practice for manufacturing joint replacements, as hip and knee implants [1,10,16].

The main steps considered for the research program are the following: materials characterization (see Section 2.2), defining the cutting regime parameters, preparation of the material samples for cutting tests, sample processing (dry end-milling), roughness measurement (Ra parameter), data registration, data processing and discussion.

The material samples were provided as round bars, with a diameter of 8 mm for the CoCrMo alloy, and a diameter of a 6.5 mm for the CoCrWNi alloy. These samples were cut in order to be used for material characterization analysis and to perform the end-milling tests. Sample preparation and materials characterization were performed in the laboratories of University Politehnica of Bucharest, and the cutting tests were performed at the Romanian Research and Development Institute for Gas Turbines—COMOTI.

### 2.1. Materials Characterization

As hardness and microstructure have major influences on material machinability, first a characterization of the Co–Cr-based alloys was performed, including chemical composition analysis, metallographic structure analysis, and hardness measurements in different areas. The chemical composition is presented in Table 1.

To highlight the metallographic aspects of the CoCrMo and CoCrWNi alloys, samples were cut using the precision cutting machine IsoMet 4000 under a coolant jet. The samples were then hot embedded (195 °C) in phenolic resin, after which they were subjected to grinding and polishing operations using a series of abrasive discs with different granulations (320 to 1000 grit) and fine abrasive powders (alumina alpha 6 to 0.1 μm diameter). The electrochemical attack was carried out in a solution of 2–10 g CrO3 in 100 mL of water at 3 volts for 10 s. Microstructure examination was performed with an Olympus GX51 microscope (Hamburg, 20034, Germany) equipped with AnalySIS image processing software (Munster, Germany, 2007, build 1274).

**Figure 1:**
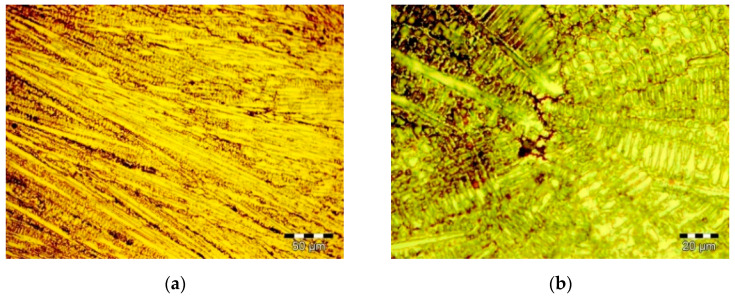
Microstructure of the CoCrMo alloy: (**a**) sample lateral area; (**b**) detail of the sample central area.

**Figure 2:**
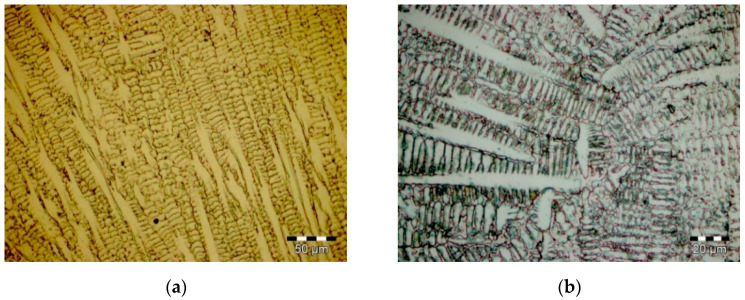
Microstructure of the CoCrWNi alloy: (**a**) sample lateral area; (**b**) detail of the sample central area.

Images of the Co–Cr alloy effective microstructures are presented in Figure 1 for the CoCrMo alloy and Figure 2 for the CoCrWNi alloy.

An analysis of the obtained microstructure images shows the following: for the CoCrMo alloy a fine columnar dendritic microstructure with Co-rich, Cr-rich and Mo-rich interdendritical phases can be identified (see Figure 1a). The CoCrWNi alloy shows a columnar dendritic microstructure with W, Co-rich, Cr-rich and Cr-Ni inter-dendritic precipitates (see Figure 2a). The detail of the central area of the CoCrWNi sample highlights large Co-rich dendrites (5–10 µm), while the CoCrMo sample core center detail highlights grain refining compared to CoCrWNi alloys, and a high tendency to accumulate secondary phases in interdendritic areas (see Figure 1b and Figure 2b).

The materials’ microhardness effective values were determined using the Vickers HV 0.5 method. Five hardness measurements were performed, in a straight line, using the microhardness tester Shimadzu HMV 2TE (Shimadzu Europa, Duisburg, 47269, Germany) from the LAMET laboratory of University Politehnica of Bucharest. The microhardness variation intervals and their mean values are shown in Table 2.

### 2.2. Cutting Experiment Setup

#### 2.2.1. Milling Equipment Description and Experimental Conditions

**Figure 3:**
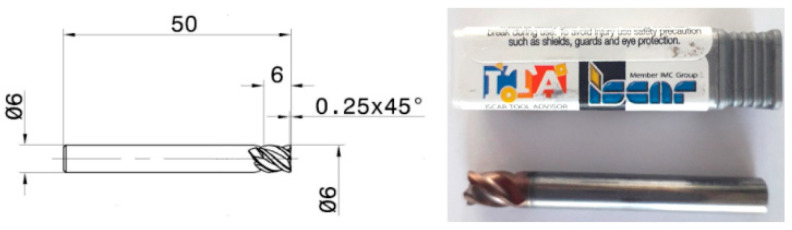
Type IC 608 endmill cutter used for performing the cutting tests, provided by ISCAR.

The cutting experiment was performed at the National Research and Development Institute for Gas Turbines—COMOTI, within the NC Programming and Technology Research Department. The machine tool considered for performing the milling tests was a DAH LIH MCV-1250B vertical machining center with a 3 NC axis system (Taichung 413, Taiwan). The main specifications/working capabilities of the machine-tool are: longitudinal travel (X): 1250 mm, cross travel (Y): 650 mm, headstock travel (Z): 700 mm, maximum spindle speed: 8000 rpm, maximum cutting feed: 10,000 mm/min, minimum input increment: 0.001 mm and spindle drive power: 7.5 kW. The cutting tool is a Ø6 endmill cutter PVD coated with AlTiCrSiN layers provided by ISCAR (Figure 3), with 4 flutes, different helixes (values of 36° and 40°) and variable pitch for chatter dampening.

**Figure 4:**
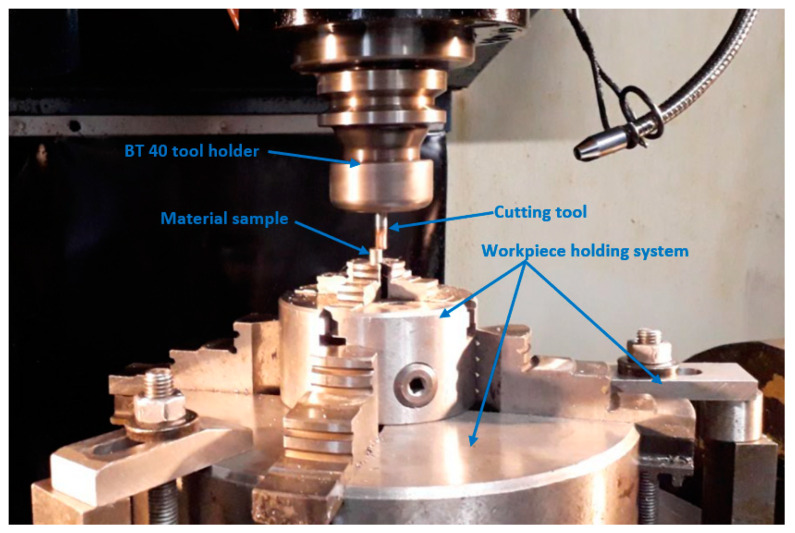
Milling technological system used for the cutting tests.

The cutting tool was fixed in the machining center’s BT 40 tool holder. The system used for holding the cutting samples consisted of a Ø144 mm 4-jaw chuck used for directly holding the cutting samples, which was secured on a Ø450 mm 4-jaw chuck fixed with clamps on the machining center table. All the elements presented in this section compose the milling technological system used for the experimental phase. The main components of this system are shown in Figure 4.

#### 2.2.2. Cutting Parameters and Experimental Plan

The cutting regime variables considered for the end-milling experiment were established, taking into account the analyzed Co–Cr based alloy properties, the cutting tool manufacturer recommendations and the capabilities of the machining center. The cutting parameters (independent variables) considered for the milling experiments were the axial depth of cut a_p_ (mm), the feed per tooth f_z_ (mm/tooth) and the cutting speed v_c_ (m/min). The radial depth of cut a_e_ (mm) was kept constant, a_e_ = 6 mm (the milling tool diameter). For each parameter, three levels were considered, as shown in Table 3. The defined experimental plan consisted of 15 experimental trials, and is presented in Table 4. This experimental plan was developed using the central composite design (CCD) method and the face centered composite design type (CCF) where α = ±1; a method that involves fewer experimental trials than the factorial experiment with good accuracy. In our case, the first 8 experimental trials proposed represent the factorial portion of the design, and the 9–14 experimental trials represent the axial points, while the last trial represents the central point. Experimental trial 15 was repeated 3 times.

#### 2.2.3. Samples Preparation

**Figure 5:**
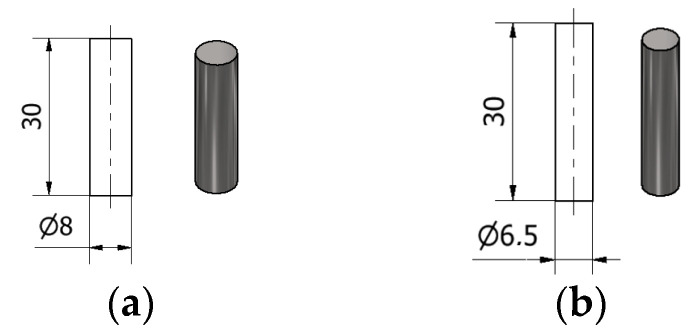
Sample dimensions: (**a**) Co-28Cr-6Mo alloy; (**b**) Co-20Cr-15W-10Ni alloy.

The samples used for the milling tests were prepared in the Cutting Technologies Laboratory of UPB. Taking into account the number of experiments that composed the defined experimental plan, a set of 15 samples was prepared for each analyzed material. The material samples were cut (part off) from rods with the diameters mentioned in the introduction of this section, with the samples’ frontal surfaces being further subjected to the end-milling process. The length of each sample was chosen to be 30 mm (Figure 5).

#### 2.2.4. Roughness Measurement Equipment and Procedure

The equipment considered for surface roughness measurement was the Insize ISR-C002 portable roughness tester with an inductive diamond tip capable of performing measurements within a range of 160 µm and an Ra resolution of 0.001 µm. The settings considered for the measurements are in accordance with ISO 4288 for Ra values between 0.1 µm and 2 µm, as follows: cut-off value λ_c_ = 0.8 mm, number of cut-offs = 5, evaluation length l_t_ = 4 mm. The surface roughness measurements were carried out across the cutting tool feed direction on three areas of each sample, and the average of these values was considered as the Ra surface value.

## 3. Results and Discussion

This section provides a presentation of the obtained results in terms of Ra roughness parameter values of the machined samples, their processing and interpretation.

### 3.1. Results Presentation

The average Ra parameter values obtained after the measurements performed on the machined samples for all experimental trials and the measurement uncertainty are presented in Table 5.

The performed measurements revealed that the Ra values belong to the following intervals: Ra ∈ [0.581, 1.463] μm for Co–20Cr–15W–10Ni, where the minimum value was registered for experiment 3 and the maximum value was registered for experiment 5; and Ra ∈ [0.491, 1.589] μm for Co–28Cr–6Mo, where the minimum value was registered for experiment 3 and the maximum value was registered for experiment 6. While looking at the Ra average values (Table 5), it can be seen that the minimum and maximum values belonged to the same experiments and the average values belong to the following intervals: Ra ∈ [0.586, 1.375] μm for Co–20Cr–15W–10Ni and Ra ∈ [0.496, 1.570] μm for Co–28Cr–6Mo. In general, the Ra values achieved for the Co-28Cr-6Mo alloy were higher than the ones achieved for Co–20Cr–15W–10Ni alloy, and this phenomenon is explained by the fact that the first sample had a higher effective hardness which made the material more difficult to cut.

For Co–20Cr–15W–10Ni, the lower average Ra roughness of 0.548 μm was achieved for a_p_ = 0.25 mm, f_z_ = 0.02 mm/tooth and v_c_ = 30 m/min and the higher average Ra roughness of 1.471 μm was achieved for a_p_ = 0.25 mm, f_z_ = 0.05 mm/tooth and v_c_ = 30 m/min, a fact that shows the high influence exerted by the feed per tooth cutting parameter on the surface quality that can be achieved. Small values of Ra roughness were also obtained for experimental trial numbers 1 (0.595 μm) and 2 (0.586 μm). If for trial2, the value obtained was explained by the lower value of f_z_ and the upper value of v_c_, for experimental trial 1, the low value at low cutting speed can be explained by the built-up edge formed on the cutting tool and the small a_p_ value.

For Co-28Cr-6Mo, the lower average Ra roughness of 0.496 μm was achieved for a_p_ = 0.25 mm, f_z_ = 0.02 mm/tooth and v_c_ = 30 m/min; and the higher average Ra roughness of 1.570 μm was achieved for a_p_ = 0.75 mm, f_z_ = 0.05 mm/tooth and v_c_ = 20 m/min. In this last case, the results were as expected, and it is known from the scientific literature that when a_p_ and f_z_ have high values and v_c_ has a small value, the value of the roughness should be high [12,26]. Small values of the Ra roughness were also obtained for experimental trials 2 (0.533 μm) and 4 (0.503 μm). If for trial 2 the value obtained was explained by the lower value of f_z_ and the upper value of v_c_ (same case as above), for experimental trial 4, the low value at low cutting speed can be explained by the built-up edge formed on the cutting tool.

**Figure 6:**
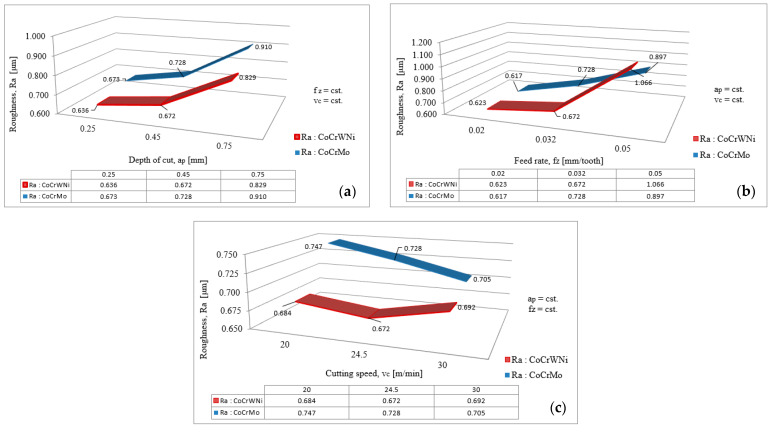
Variation of Ra roughness in relation to the milling process variables: (**a**) variation of surface roughness, Ra, in relation to the axial depth of cut, a_p_; (**b**) variation of surface roughness, Ra, in relation to the feed rate per tooth, f_z_; (**c**) variation of surface roughness, Ra, in relation to the cutting speed, v_c_.

The variation of the Ra roughness in relation with the milling process variables considered for the experimental research: axial depth of cut, a_p_; feed rate per tooth, f_z_; and cutting speed, v_c_; when two of these parameters are kept constant, is shown in Figure 6.

Analyzing the variation graphs presented in Figure 6, it can be ascertained that, in terms of constant values for the other process variables taken into account for the experiments, the highest influence on the Ra surface parameter was given by feed per tooth and the lowest influence was given by the cutting speed.

From the interdependence graphs between the Ra roughness parameter and the depth of cut (Figure 6a), when the other two parameters are constant, it can be observed that when the depth of cut increases by 80%, the Ra parameter increases by 8.17% for Co–28Cr–6Mo and by 5.66% for Co–20Cr–15W–10Ni, and when the depth of cut increases by 200%, the Ra parameter increases by 35.22% for Co–28Cr–6Mo and by 30.35% for Co–20Cr–15W–10Ni. Theoretically, this variation of the roughness values is as expected, because at a low depth of cut the roughness should have a small value, and at a high depth of cut, the roughness should have a high value; and the same dependence is expected for the feed per tooth variation [24,25,26].

From the interdependence graphs between the Ra roughness parameter and the feed per tooth (Figure 6b), when the other two parameters are constant, it can be observed that when the feed per tooth increases by 60%, the Ra parameter increases by 17.99% for Co-28Cr–6Mo and by 7.87% for Co–20Cr–15W–10Ni, and when feed per tooth increases by 150%, the Ra parameter increases by 45.38% for Co–28Cr–6Mo and by 71.10% for Co–20Cr–15W–10Ni. It can be seen that, even if the evolution of the roughness is as expected, its value increases by a higher percent in the second interval for the Co–20Cr–15W–10Ni, a fact that can be explained by tool wear.

From the interdependence graphs between the Ra roughness parameter and the cutting speed (Figure 6c), when the other two parameters are constant, it can be observed that when the cutting speed increases by 22.5%, the Ra parameter decreases by 2.54% for Co–28Cr–6Mo and by 1.75% for Co–20Cr–15W–10Ni and when the cutting speed increases by 50%, the Ra parameter decreases by 5.62% for Co–28Cr–6Mo and increases by 1.17% for Co–20Cr–15W–10Ni. As presented above, a high cutting speed should result in a low roughness and a low cutting speed should result in a high roughness of the machined surface; the fact that for Co–20Cr–15W–10Ni a higher roughness value is obtained for v_c_ = 30 m/min than for v_c_ = 20 m/min can be explained by the built-up edge formed on the cutting tool or by tool wear.

### 3.2. Prediction Model Development

Generally, roughness prediction for milling process can be done using several methods. In our research, taking into account that prediction of the Ra should be made based on 3 independent variables: axial depth of cut—a_p_ (mm), feed per tooth—f_z_ (mm/tooth) and cutting speed—v_c_ (m/min), statistical techniques were applied first to achieve Ra prediction models.

Equations (1)–(6) present the Ra roughness prediction models obtained by applying multiple linear regression (MLR), response-surface regression (RSR) and nonlinear regression(NR) for the Co-28Cr-6Mo and Co-20Cr-15W-10Ni alloys, respectively.

Ra__CoCrMo_ = 0.524344535971431 − 0.0136101606385584∗a_p_ + 22.1355978517912∗f_z_ − 0.0172205349781354∗v_c_ (1)

Ra__CoCrWNi_ = 0.0789015828122097 − 0.257581853702271∗a_p_ + 18.6487680467611∗f_z_ + 0.00916261206257762∗v_c_ (2)

Ra__CoCrMo_ = 0.8005121 − 0.5931409∗a_p_ + 44.54829∗f_z_ − 0.06556462∗v_c_ + 1.855399∗a_p_^2^ + 40.42375∗f_z_^2^ + 0.002083401∗v_c_^2^ − 3.427583∗a_p_∗f_z_ − 0.04793371∗ a_p_∗v_c_ − 0.9592412∗f_z_∗v_c_ (3)

Ra__CoCrWNi_ = 1.377755 − 0.5671411∗a_p_ − 23.55592∗f_z_ − 0.0365608∗v_c_ + 0.8959987∗a_p_^2^ + 454.1539∗f_z_^2^ − 0.0001372941∗v_c_^2^ − 37.3593∗a_p_∗f_z_ + 0.02708041∗ a_p_∗v_c_ + 1.132445∗f_z_∗v_c_ (4)

Ra__CoCrMo_ = 127.086071087871 ∗ a_p_^−0.0283522477878498^ ∗ f_z_ ^0.912892216393497^ ∗ v_c_^−0.611403498322333^ (5)

Ra__CoCrWNi_ = 3.61413779230374 ∗ a_p_^−0.228933901981133^ ∗ f_z_ ^0.827767771694901^ ∗ v_c_^0.34954784648001^ (6)

The performance of the prediction models in this paper was examined based on their coefficient of determination (R^2^ value) between the output (predicted) values and the target (experimental) values for each alloy. The value of the coefficient of determination (R^2^) is a statistical measurement of how the regression model matches with the real data points. For a perfect match fit of data, the regression model should have a coefficient of determination of 1. The R^2^ values obtained for the presented models are shown in Table 6 and Table 7.

Based on results from Table 6 for Co–28Cr–6Mo, the best result was achieved for the model obtained by response-surface regression analysis (R^2^ = 0.8561), and the weakest result by multiple linear regression (R^2^ = 0.7650). Similar results were obtained for the Co-20Cr–15W-10Ni alloy where the best and weakest results were achieved for the same models (R^2^ = 0.93394 for RSR and R^2^ = 0.7026 for MLR). However, even if the achieved R^2^ values for RSR models indicate that they can characterize the process, being close enough to the desired value of 1, especially for Co–20Cr–15W–10Ni, a prediction model that can better characterize the relationship between variables is desirable.

In this context, other prediction models were investigated, and it was determined that the model based on an artificial neural network (ANN) can produce better prediction results in case of nonlinear relationships between the dependent and independent variables.

The development process of the proposed ANN model and its results for the two analyzed Co–Cr alloys will be further presented.

#### 3.2.1. Artificial Neural Network (ANN)

**Figure 7:**
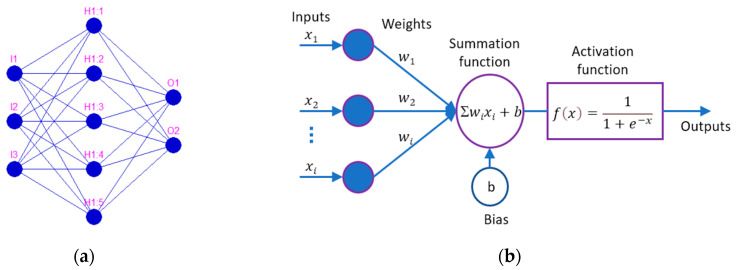
Artificial neural network (ANN): (**a**) ANN—general architecture; (**b**) mathematical representation of an artificial neuron.

An artificial neural network (ANN) is a computational system that uses a network of functions (nodes) to process data input into a desired output. It was inspired by the biological nervous system; therefore, the network is composed of interconnected elements (nodes) called artificial neurons [21,59,60]. The ANN computational system consists of three layers: the input layer, which contains input neurons that represent input data; the hidden layer, which contains a specific number of hidden neurons; and the output layer, which consists of the output data (the number of neurons is equal to the number of desired outputs). In Figure 7a, the input layer contains 3 neurons: I1, I2, I3, the hidden layer contains 5 neurons: H1:1, H1:2, H1:3, H1:4, H1:5 and the output layer contain 2 neurons: O1 and O2. A general mathematical representation of an individual neuron within an ANN is represented in Figure 7b. The input vector (x_1_,x_2_,…x_i_) is transferred using a connection that multiplies its strength by weights (w_i_). The output is obtained using a summation function with a specific bias (b) and an activation function. This study used a feedforward neural network with a sigmoidal unipolar activation function [61].

The teaching algorithm chosen for the ANN study was resilient propagation (RProp) due to its universality and because it adapts the step size dynamically for each weight independently [62].

#### 3.2.2. ANN Application Software for Prediction of Surface Roughness

The present ANN study attempted to predict the surface roughness Ra (µm) for the dry milling process for two alloys (Co–20Cr–15W–10Ni and Co–28Cr–6Mo) based on three process parameters (independent variables): axial depth of cut, a_p_ (mm), feed per tooth, f_z_ (mm/tooth), and cutting speed, v_c_ (m/min).

An ANN application was developed in LabVIEW, a system engineering software that allows use of a graphical programming approach for developing data analysis algorithms and custom engineering user interface design. A specific addon toolkit was also used: NI Super Simple Neural Network, which is based on a feedforward neural network with a sigmoidal unipolar activation function and uses the RProp teaching algorithm [63].

**Figure 8:**
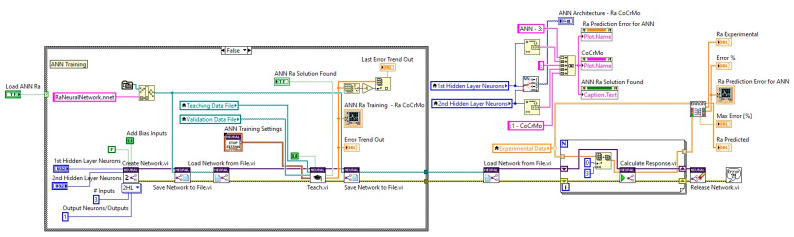
Partial diagram of application software to create, save, teach, test and save an ANN.

Drawing upon the experimental data sets of the analyzed Co–Cr alloys, an algorithm based on specific Sub-VIs for preparing the data for the ANN training process was developed, generating customized teaching and validation data files. The use of different data sets for teaching, validation, and testing was considered optimal and implemented. A partial diagram of the code to create, save, teach, test and save an ANN developed in LabView is presented in Figure 8.

Because the experimental data sets contain three Ra measurements taken in different areas for each trial, the ANN data set was prepared accordingly to prevent retraining.

**Figure 9:**
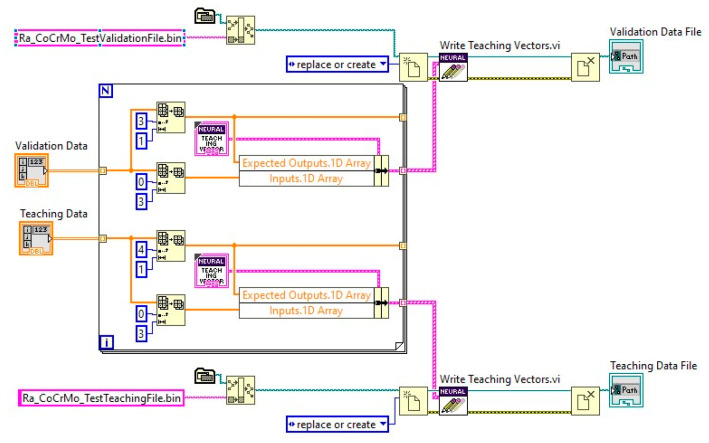
Partial diagram of application software for preparing the teaching and validation data files of an ANN.

Based on experimental data sets the ANN was trained by using a different training data set after normalizing the data. Figure 9 shows how different data files were generated using dedicated sub-VIs to produce the teaching and validation data files.

For the test data, another set of data was used, which was different from the teaching and validation data files.

A specific searching algorithm for generating and training different ANN architectures with two hidden layers was designed. For each alloy, based on its experimental data set, the algorithm generated architectures with a different number of neurons for each hidden layer, with the number of neurons varying between 3 and 90.

For each ANN configuration, an architecture with a set of neurons for each hidden layer was generated and trained using the specific sub-VI RProp teaching algorithm. Each time when, in the training process, an ANN solution was found (the ANN error trend out became less than the error goal) then the resulting ANN was saved, and its prediction was tested with another experimental data set. The max error (%) and coefficient of determination R^2^ were calculated. In all cases when a solution was found, the ANN architecture was automatically saved.

The searching algorithm was executed for each data set for Co–20Cr–15W–10Ni and Co–28Cr–6Mo using different ANN training setting parameters which were varied between the following ranges:

- Max epocs: 8000–20,000;
- Error goal: 0.2–0.1;
- Max time (s): 15–40 s;
- Error evaluation: over both sets; and
- Vectors per iteration: 200–400.

**Figure 10:**
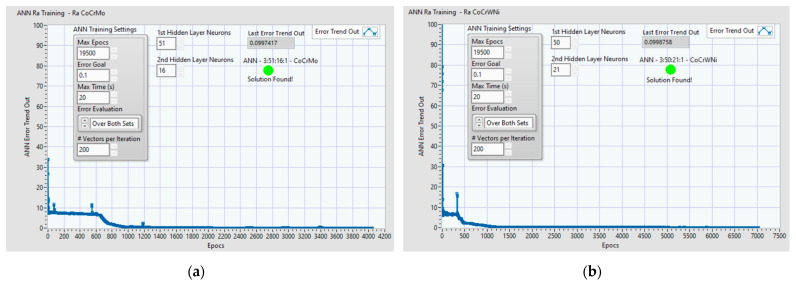
Example of ANN training process: plot of error trend out and other relevant parameters: (**a**) ANN 3:51:16:1 for Co–28Cr–6Mo; (**b**) ANN 3:50:21:1 for Co–20Cr–15W–10Ni.

Figure 10 presents the plot of the training ANN process together with other relevant parameters: ANN training settings: error goal, max time, training error evaluation, vectors per iteration. The training process is validated or invalidated by the algorithm, which is indicated by the green LED which displays “Solution was found” and indicates the last value of “Error trend out” of the teaching process. If the process ends with “Solution found,” then the corresponding ANN data files are saved (Example Figure 10a: RaNN_CoCrMo_51 16Error0.3.nnet, Ra_CoCrMo_TestTeachingFile51 16Error0.bin and Ra_CoCrMo_TestValidationFile51 16Error0.bin).

#### 3.2.3. ANN Analysis and Prediction

**Figure 11:**
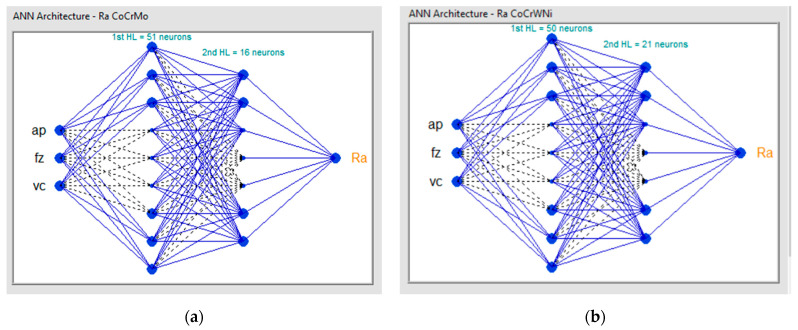
ANN architectures: (**a**) ANN 3:51:16:1 for Co-28Cr-6Mo; (**b**) ANN 3:50:21:1 for Co–20Cr–15W–10Ni.

Based on the search algorithm, after an ANN was found and saved, the performance of prediction was tested with another set of experimental data. In Figure 11 examples with the resulting ANN architectures which were found suitable for our two alloys are displayed.

**Figure 12:**
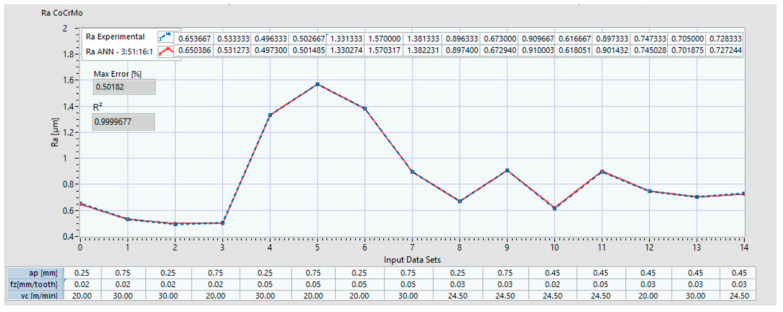
Ra comparison between experimental data and ANN predictions with architecture 3:51:16:1 for Co–28Cr–6Mo.

**Figure 13:**
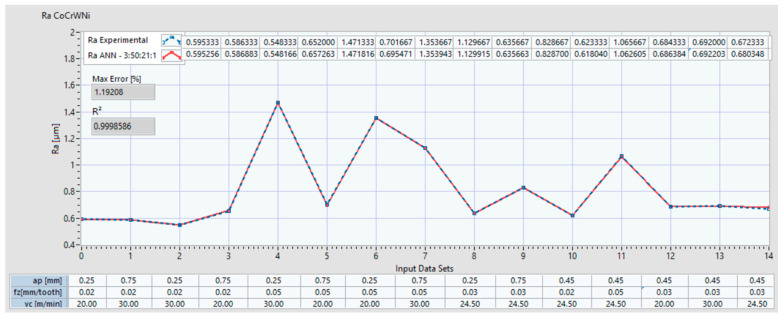
Ra comparison between experimental data and ANN predictions with architecture 3:50:21:1 for Co–20Cr–15W–10Ni.

For the Co-28Cr-6Mo alloy, Figure 12 indicates the two plots of the Ra: one is for the experimental data set, and the other is the data set with the prediction which resulted from the ANN (ex: ANN 3:51:16:1 is the set of prediction results for an ANN with 3 inputs, 51 neurons on the 1^st^ hidden layer and 16 neurons on the 2^nd^ hidden layer). The coefficient of determination R^2^ and the maximum error (%) of the predicted ANN data set compared with the experimental set is also indicated. In the lower part of the plot, the input data sets are presented. Figure 13 shows the plots of the Ra with the experimental data and with the predictions that resulted from the ANN 3:50:21:1 for Co–20Cr–15W–10Ni.

**Figure 14:**
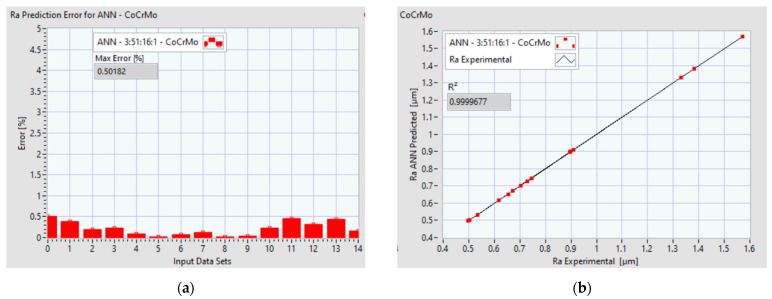
Ra prediction performance of ANN 3:51:16:1 for Co–28Cr–6Mo: (**a**) error distribution per data set; (**b**) coefficient of determination R^2^ and = correlation between the predicted values and entire data.

**Figure 15:**
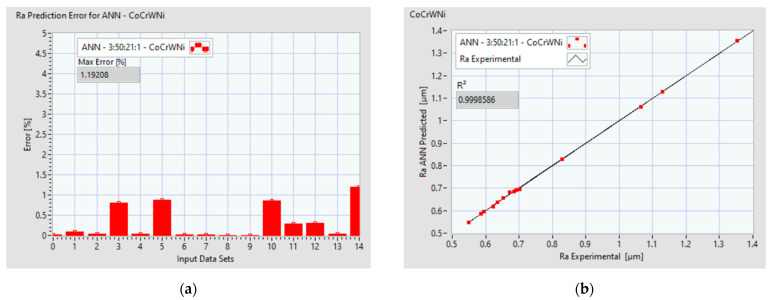
Ra prediction performance of ANN 3:50:21:1 for Co–20Cr–15W–10Ni: (**a**) error distribution per data set; (**b**) coefficient of determination R^2^ and correlation between the predicted values and entire data.

Figure 14a and Figure 15a display the performance of the ANN, where the prediction error of Ra is presented for each data set. Figure 14b and Figure 15b indicate the coefficients of determination R^2^ and the correlation between the predicted values and entire data set.

**Figure 16:**
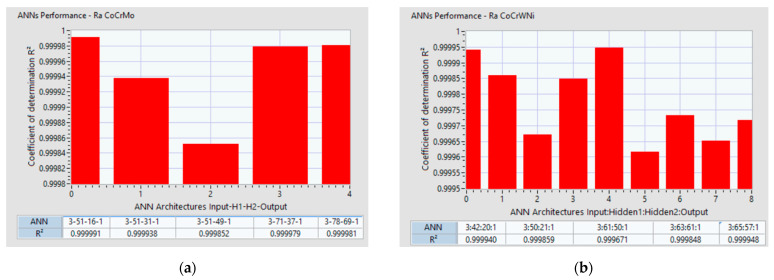
Coefficient of determination variation with ANN layers: (**a**) configuration for Co–28Cr–6Mo alloy; (**b**) configuration for Co–20Cr–15W–10Ni.

The present study succeeded in identifying and analyzing several ANN architectures which can adhere to the initial goal of a less than 5% prediction error and a coefficient of determination R^2^ greater than 0.9996. Figure 16a shows 5 ANN architectures which were in line with these requirements for the Co–28Cr–6Mo alloy. Figure 16b shows 9 ANN architectures which were in line with these requirements for the alloy Co-20Cr-15W-10Ni.

It can be confirmed that there are no general rules for defining the number of hidden layers for all ANN architectures and this can be determined only by employing a trial-and-error method [64]. The important factor is the number of nodes for each layer, and it was proven that the search algorithm developed in the present research succeeded in identifying different ANN architectures with different node distributions for each hidden layer. For the Co–28Cr–6Mo alloy, ANN 3-51-16-1 had the best coefficient of determination R^2^ = 0.999991 while having an architecture with 3 inputs (a_p_, f_z_, v_c_), 51 neurons on the 1st hidden layer, 16 neurons on the 2nd hidden layer, and one output R_a_. For the Co–20Cr–15W–10Ni alloy, ANN 3-61-50-1 has the best coefficient of determination R^2^ = 0.999671, with an architecture with 3 inputs (a_p_, f_z_, v_c_), 61 neurons on the 1st hidden layer, 50 neurons on the 2nd hidden layer, and one output Ra.

For a better understanding of the roughness prediction behavior during milling process of the selected ANNs architectures, another analysis was conducted where some input datasets were extended.

**Figure 17:**
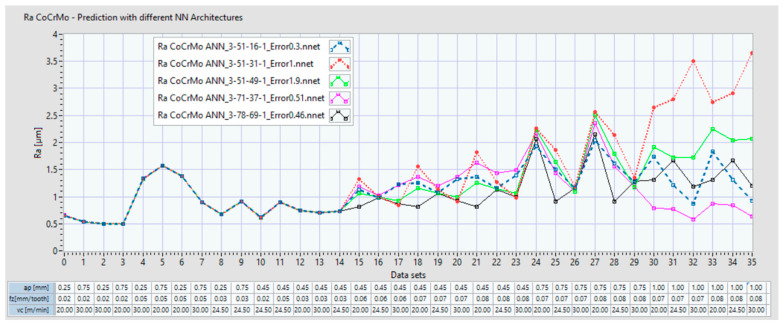
Prediction of Ra with 5 ANNs for Co–28Cr–6Mo with a data set of 36 inputs.

Figure 17 displays the prediction results for five ANNs found for the milling process of the Co–28Cr–6Mo alloy which were in line with performance prediction requirements for the initial range of input data sets. In this case, the input data set was extended from 15 to 36 with additional data corresponding to the following extending limits:

- a_p_ (mm): maximum limit was extended from 0.75 mm to 1 mm;
- f_z_ (mm/tooth): maximum limit was extended from 0.05 to 0.08;
- v_c_ (m/min): maximum limit was not extended.

**Figure 18:**
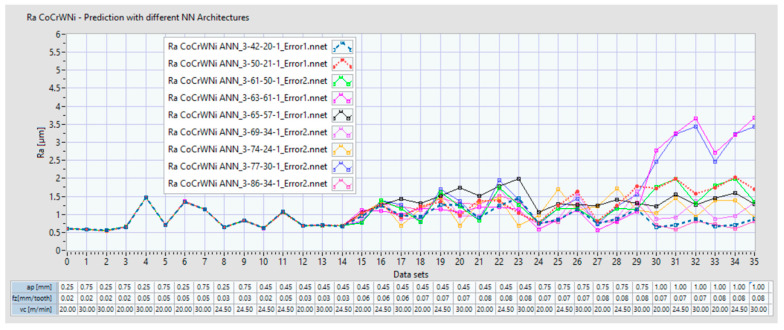
Prediction of Ra with 10 ANNs for Co-20Cr-15W-10Ni with a data set of 36 inputs.

Figure 18 displays the prediction results with the same 36 input data set for nine ANNs found for the milling process of the alloy Co-20Cr-15W-10Ni.

The results indicate that for Co–28Cr–6Mo alloy, starting with the data set 23, the Ra exceeded 2 μm. This occurs when a_p_ exceeds 0.75 mm, f_z_ is 0.07 mm/tooth and 0.08 mm/tooth, and v_c_ is 20, 24.5 or 30 m/min. A first interpretation is that an Ra of less than 2 μm is difficult to obtain from milling with an a_p_ higher than 0.75 mm.

The ANN prediction behavior starting with data set 29 became uncertain and varied from one ANN architecture to another. Consequently, this opened another research direction towards performing more experimental trials where milling process parameters should fall within the following extended ranges: a_p_ (0.75 to 1) mm, f_z_ 0.07 mm/tooth and 0.08 mm/tooth, and v_c_ 20, 24.5, or 30 m/min.

For the Co–20Cr–15W–10Ni alloy, the Ra seems to exceed 2 μm starting with data set 29 for 2 of ANNs. Additionally, it can be observed that the ANN prediction behavior starting with data set 29 became uncertain and varied. We may conclude that when we increase the a_p_ over 0.75 mm, either the Ra may exceed 2 μm or the prediction is not accurate.

The ANN application software designed for this study is based on an architecture based on sub-Vis which can be extended and integrated in larger subsystems to cover predictive and adaptive roles in the design process of future products made from materials such as these alloys.

Another direction of software development is to build ANN models based on more relevant input process parameters (independent variables) such as displacement of tool vibration, tool wear, etc. Increasing the number of variables as an input to an ANN may increase the accuracy of the predicted answers.

## 4. Conclusions

Ra roughness is an important parameter in the evaluation of the quality of a machined surface. Consequently, developing prediction models for this parameter is important for improving the performance of the machining operation, product functionality and optimizing costs. Even if end-milling is not always the last operation performed in order to obtain the final characteristics prescribed for a certain surface, especially in regard to medical implants, obtaining small roughness values during this process will reduce the cost and increase the performance of the finishing operations.

In the present study, ANN models were used to predict the surface roughness for the dry end-milling of the biomedical alloys Co–20Cr–15W–10Ni and Co–28Cr–6Mo. The models used three input process parameters (independent variables): axial depth of cut, a_p_ (mm); feed per tooth f_z_ (mm/tooth); and cutting speed v_c_ (m/min), which were initially measured in an experimental design. An ANN application for surface roughness prediction was developed based on the obtained data.

The relevant conclusions associated with the research presented in this paper are as follows:

- The obtained experimental data shows that the Ra values that can be obtained by dry end-milling with an AlTiCrSiN PVD coated tool for Co–28Cr–6Mo and Co–20Cr–15W–10Ni alloys used in biomedical applications are under 2 μm. Consequently, the finishing operation necessary to obtain the final surface quality will have a smaller cost generated by a shorter processing time and, implicitly, a lower usage of the finishing cutting tool.
- When maintaining two of the considered process variables at a constant value, it can be observed that the Ra values obtained for machining Co–20Cr–15W–10Ni were predominantly smaller than those obtained for Co–28Cr–6Mo.
- The results obtained via regression analysis models for both alloys indicated less accurate prediction of Ra compared with the ANN models.
- The comparison of the measured results to the results originating from the numerical simulation indicated that the ANN model allows for the accurate estimation of the roughness value of the surface processed by milling, consequently becoming a valuable tool for technical applications. The generation of several ANN architectures with high prediction performance may lead to further studies and research efforts, which may include other process parameters and may help in establishing a correlation between machining processes and the processing requirements of the medical implants.
- Developing customized software for the prediction of Ra based on ANNs could be a development path to investigate for a future generation of applications which could assist the design process of implants for medical applications. Increasing the number of relevant input process parameters to the ANN may increase the accuracy of the predicted answer for Ra.
- To obtain an Ra value of less than 2 μm for the Co–20Cr–15W–10Ni or Co–28Cr–6Mo alloys, the study showed that the axial depth of cut a_p_ should not exceed 0.75 mm, the feed per tooth f_z_ should be 0.07 mm/tooth and 0.08 mm/tooth and the v_c_ should be 20, 24.5, or 30 m/min.
- The presented results are in line with the concept of vertical integration, applying Industry 4.0 concepts and principles and may lead to new directions for developing useful ANN submodule tools which can assist the concept designing process of future medical implants based on biomedical alloys.

## Figures and Tables

**Table 1:** Chemical composition of the CoCrMo and CoCrWNi alloys.

Material	Chemical Elements (%)										
	Cr	Mo	Ni	Fe	C	Si	Mn	W	P	S	Co
Co–28Cr–6Mo	27.8	5.65	2.08	0.39	0.27	0.69	0.75	0.14	0.02	0.01	Bal.
Co–20Cr–15W–10Ni	19.68	-	10.13	2.11	0.09	0.72	1.04	15.1	0.015	0.022	Bal.

Bal.: Balanced element.

**Table 2:** CoCrMo alloy and CoCrWNi alloy microhardness effective values.

Material	Hardness Type	Microhardness Measurements					Mean Value
Co–28Cr–6Mo	HV 0.5	431	475	467	456	450	456
	HRC	43.7	47.3	46.6	45.8	45.3	45.7
Co–20Cr–15W–10Ni	HV 0.5	370	366	345	351	345	355
	HRC	37.7	37.3	35	35.6	35	36.1

**Table 3:** Cutting process parameters (variables) and their considered levels.

Cutting Parameter	Symbols	Levels		
		−1	0	+1
Axial depth of cut (mm)	a_p_	0.25	0.45	0.75
Feed per tooth (mm/tooth)	f_z_	0.02	0.032	0.05
Cutting speed (m/min)	v_c_	20	24.5	30

**Table 4:** Experimental plan.

Experiment No.	Process Variables Values		
	Axial Depth of Cut, a_p_ (mm)	Feed per Tooth, f_z_ (mm/tooth)	Cutting Speed, v_c_ (m/min)
1	0.25	0.02	20
2	0.75	0.02	30
3	0.25	0.02	30
4	0.75	0.02	20
5	0.25	0.05	30
6	0.75	0.05	20
7	0.25	0.05	20
8	0.75	0.05	30
9	0.25	0.032	24.5
10	0.75	0.032	24.5
11	0.45	0.02	24.5
12	0.45	0.05	24.5
13	0.45	0.032	20
14	0.45	0.032	30
15	0.45	0.032	24.5

**Table 5:** Ra average values for the Co–Cr alloys analyzed.

Experiment No.	Process Variables Values			Ra Average Values ± u ^1^ (μm)	
	a_p_ (mm)	f_z_ (mm/tooth)	v_c_ (m/min)	Co-20Cr-15W-10Ni	Co-28Cr-6Mo
1	0.25	0.02	20	0.595 ± 0.003	0.654 ± 0.026
2	0.75	0.02	30	0.586 ± 0.004	0.533 ± 0.016
3	0.25	0.02	30	0.548 ± 0.004	0.496 ± 0.032
4	0.75	0.02	20	0.652 ± 0.004	0.503 ± 0.034
5	0.25	0.05	30	1.471 ± 0.025	1.331 ± 0.006
6	0.75	0.05	20	0.702 ± 0.015	1.570 ± 0.017
7	0.25	0.05	20	1.354 ± 0.014	1.381 ± 0.041
8	0.75	0.05	30	1.130 ± 0.014	0.896 ± 0.087
9	0.25	0.032	24.5	0.636 ± 0.016	0.673 ± 0.024
10	0.75	0.032	24.5	0.829 ± 0.024	0.910 ± 0.008
11	0.45	0.02	24.5	0.623 ± 0.005	0.617 ± 0.009
12	0.45	0.05	24.5	1.066 ± 0.006	0.897 ± 0.025
13	0.45	0.032	20	0.684 ± 0.011	0.747 ± 0.042
14	0.45	0.032	30	0.692 ± 0.003	0.705 ± 0.018
15	0.45	0.032	24.5	0.672 ± 0.039	0.728 ± 0.034

^1^ u = expanded measurement uncertainty.

**Table 6:** Comparison of coefficient of determination with different regression models for Co–28Cr–6Mo alloy.

Model (Equation)	Type of Regression	Coefficient of Determination R^2^
Equation (1)	Multiple linear regression	0.7650
Equation (3)	Response-surface regression	0.8561
Equation (5)	Nonlinear regression	0.793799

**Table 7:** Comparison of coefficient of determination with different regression models for Co–20Cr–15W–10Ni alloy.

Model (Equation)	Type of Regression	Coefficient of Determination R^2^
Equation (2)	Multiple linear regression	0.7026
Equation (4)	Response-surface regression	0.93394
Equation (6)	Nonlinear regression	0.770015

## Data Availability

The data and all related information presented in this study are available on request from the corresponding authors.
